# High expression levels of COX-2 and P300 are associated with unfavorable survival in laryngeal squamous cell carcinoma

**DOI:** 10.1007/s00405-012-2275-1

**Published:** 2012-11-24

**Authors:** Yan-Feng Chen, Rong-Zhen Luo, Yong Li, Bo-Kang Cui, Ming Song, An-Kui Yang, Wen-Kuan Chen

**Affiliations:** 1 State Key Laboratory of Oncology in South China, Department of Head and Neck Surgery, Sun Yat-sen University Cancer Center, Guangzhou, Guangdong People’s Republic of China; 2 State Key Laboratory of Oncology in South China, Department of Pathology, Sun Yat-sen University Cancer Center, Guangzhou, Guangdong People’s Republic of China; 3 State Key Laboratory of Oncology in South China, Department of Hepatobiliary Oncology, Sun Yat-sen University Cancer Center, Guangzhou, Guangdong People’s Republic of China

**Keywords:** Laryngeal squamous cell carcinoma (LSCC), Prognosis, Survival, P300, COX-2

## Abstract

In order to provide a basis for clinical treatment decisions, we explored whether there was a correlation between the expression of COX-2 and P300 and clinical factors in a group of patients with laryngeal squamous cell carcinoma (LSCC). A retrospective analysis of clinicopathological data was conducted in 80 patients with LSCC who presented between January 1997 and December 1998. An immunohistochemistry tissue microarray was conducted of 80 surgically resected LSCC and 20 adjacent normal tissue specimens. Survival analysis and Kaplan–Meier curves were used to compare the effects of clinicopathological factors on survival. The Cox model was applied for multivariate analysis. The expression level of COX-2/P300 in LSCC tissues and adjacent normal tissues were 47.5/50.0 versus 0.0/15.0 %. The expression of COX-2 and P300 was correlated with higher T category, N category, clinical staging, histological grade and recurrence (*P* < 0.05). P300 expression was correlated with COX-2 expression (*P* < 0.05). Univariate survival analysis showed that P300, COX-2, N category, clinical staging and recurrence factors were closely correlated with unfavorable survival (*P* < 0.05). Multivariate analysis showed that COX-2 expression, histological grade and recurrence were independent prognostic factors for LSCC. High expression levels of COX-2 and P300 indicated poor survival outcomes for patients with LSCC.

## Introduction

Laryngeal carcinoma is a common head and neck tumor. The larynx is an important organ for pronunciation, breathing and swallowing and patients with laryngeal carcinoma can develop dysphonia, dysphagia and dyspnea. To achieve the best chance of a cure, the larynx is usually sacrificed during the surgical management of laryngeal squamous cell carcinoma (LSCC), and complete laryngectomy is performed for advanced stage cases with consequent radical changes to physiological and psychological function. Within the past 20 years, surgical techniques have improved, and combined radio- and chemotherapy offers the opportunity to preserve the larynx. However, the survival rate for advanced stage cases remains low. Further research on the molecular biology mechanisms that underlie LSCC has great importance in improving outcomes for this disease.

There are two cyclooxygenase (COX) enzymes, COX-1 and COX-2. COX-1 is a housekeeping gene that is expressed constitutively in most tissues [1]. In contrast, COX-2 is an immediate-early response gene that is induced by a variety of stimuli [2]. Multiple studies have confirmed that high expression levels of COX-2 were closely related to the development and prognosis of many tumors [3–5].

P300 proteins play a central role in the regulation of gene transcription. They are transcriptional coactivators that can integrate multiple signal-induced pathways and coordinate gene expression, acting as crucial scaffolds for the formation of transcriptional initiation complexes [6]. The oncogenic role of P300 has been reported in lung, colorectal, breast and prostate cancers, and its overexpression is indicative of a poor prognosis [7–10]. A recent study has shown that changes in the expression of P300 are associated with esophageal squamous cell carcinoma (ESCC) invasion and metastasis in vitro [11]. However, the significance of P300 expression for the prognosis of LSCC has not been elucidated. P300 is closely correlated with variety of acetylated gene promoters. Some studies have shown that P300 is related to the acetylation of the COX-2 gene promoter. Therefore, the regulation of P300 expression could inhibit COX-2 expression and consequent tumorigenesis [12].

Our study retrospectively investigated the role of P300 and COX-2 expression in LSCC with the use of a tissue microarray method and analyzed the correlation between the expression of these proteins and clinicopathological features. This study would help to provide a theoretical basis for the further clinical prevention and treatment of LSCC.

## Materials and methods

### Clinical data

The inclusion criteria for cases were as follows: (1) patients hospitalized with laryngeal cancer in Sun Yat-sen university cancer center between January 1997 and December 1998; (2) the pathological diagnosis was squamous cell carcinoma, including well, moderately and poorly differentiated tumors; (3) radical surgery was performed for cancer treatment, including partial/total laryngectomies and neck dissection; (4) the patients did not receive associated radiotherapy, chemotherapy or surgery prior to their hospitalization; (5) the paraffin section was well preserved and (7) integrated follow-up data were available.

All clinical data from the 80 cases with LSCC are summarized in Table 1. All cases were restaged according to the American Joint Committee on Cancer (AJCC) TNM Staging System for the Larynx (7th ed., 2010). Tumor size and the presence of neck node metastases were determined by clinical, electronic fiber laryngoscopy and radiological examinations, including CT and MRI scans. The possible presence of distant metastasis was assessed by chest X-ray films, bone scintigraphy or ultrasound examinations.

**Table 1:** The relationship between the expression of COX-2 and P300 and clinicopathological factors

Factor	COX-2				P300			
	+	−	RR	*P*	+	−	RR	*P*
Gender								
Male	35	40	0.065	0.569	37	38	0.052	0.649
Female	3	2			3	2		
Age								
<60 years	21	21	0.025	0.787	22	20	0.021	0.856
≥60 years	17	21			18	20		
Histology grade								
Well differentiated	11	28	0.396	**0.000**	13	26	0.349	**0.001**
Moderately differentiated	16	11			16	11		
Poorly differentiated	11	3			11	3		
T category								
T1 + T2	20	33	0.300	**0.007**	22	31	0.258	**0.021**
T3 + T4	18	10			18	9		
N category								
N0	28	41	0.343	**0.002**	30	39	0.323	**0.003**
N+	10	1			10	1		
Clinical staging								
I + II	18	32	0.297	**0.007**	20	30	0.258	**0.021**
III + IV	20	10			20	10		
Anatomic subsite of tumor								
S	12	4	0.275	**0.013**	10	6	0.125	0.269
G	36	28			30	34		
Recurrence								
Yes	19	4	0.447	**0.000**	18	5	0.359	**0.001**
No	19	38			22	35		
P300								
+	33	7	0.701	**0.000**				
−	5	35						

Bold values indicate statistical significance (*P* < 0.05)

*S* superglottic, *G* glottic

### Tissue microarray construction

Tumor tissue samples from 80 cases were collected, fixed in formalin\, and embedded in paraffin. Hematoxylin and eosin-stained slides were reviewed by two senior pathologists to define representative tumor regions. Two targeted core samples of each specimen were obtained using a tissue array instrument (MiniCore instruments; Alphelys, Plaisir, France).

Tissue cylinders with a diameter of 10 mm were punched and arrayed on a recipient paraffin block. Sections (5 μm) of the tissue array (recipient) block were cut and placed on glass slides. After the exclusion of cores with inadequate tissue after sectioning and tissue transfer, the final immunohistochemical analyses included core biopsies from 80 LSCC cases. Among the 80 cases, 20 cases of formalin-fixed paracancerous normal laryngeal tissues, which served as controls, were included according to the method described above.

### Immunohistochemistry and assessment

Immunohistochemical staining was performed using the tissue microarray sections that were rehydrated by means of a graded alcohol series. Endogenous peroxidase activity was blocked with 0.3 % hydrogen peroxide for 15 min. For antigen retrieval, the tissue microarray slides were boiled in Tris (hydroxymethyl) aminomethane-EDTA buffer (pH 8.0) in a pressure cooker for 20 min. Nonspecific binding was blocked with 10 % normal goat serum for 20 min. The tissue microarray slides were incubated with mouse monoclonal anti-KAT3B/P300 antibody (ab54984, 3 μg/mL dilution; Abcam, Cambridge, MA, USA) and mouse monoclonal anti-COX-2 antibody (sc-166475, 1:120, Santa Cruz Biotechnology, Inc.) for 12 h at 4 °C in a moist chamber. Subsequently, the slides were sequentially incubated with biotinylated rabbit anti-mouse immunoglobulin antibody at a concentration of 1:100 for 30 min at 37 °C and then with a streptavidin-peroxidase conjugate for 30 min at 37 °C with diaminobenzidine as the chromogen substrate. The nucleus was counterstained using Meyer’s hematoxylin. The negative control was obtained by replacing the primary antibody with PBS.

**Fig. 1:**
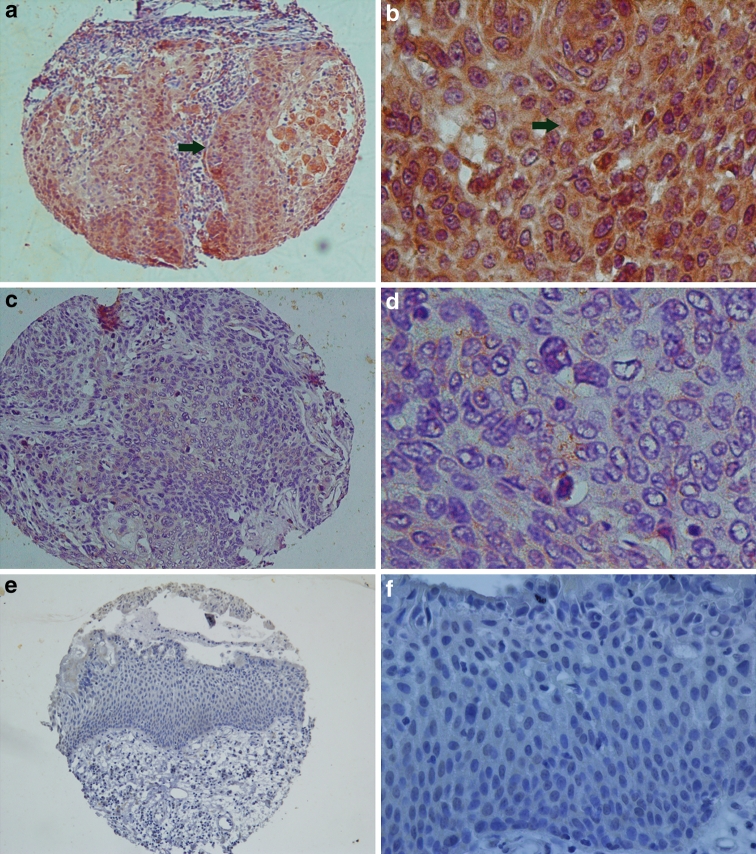
COX-2 expression according to immunohistochemical staining of LSCC samples. **a**, **b** A laryngeal squamous cell carcinoma (LSCC) case demonstrating a high expression level of COX-2 (*arrow*) detected in the cytoplasm of carcinoma cells (magnification: **a** ×100; **b** ×400); **c**, **d** Negative expression level of COX-2 detected in LSCC (magnification: **c** ×100; **d** ×400); **e**, **f** Negative expression level of COX-2 detected in adjacent normal tissue of LSCC (magnification: **e** ×100; **f** ×400)

All slices were reviewed by two experienced pathologists independently. Information on cell shape, atypia, interstitial constituents and the invasion of surrounding tissues was collected. The assessment of immunohistochemical stains of the tissue microarray slices were interpreted as either positive or negative; more than 10 % of cells that stained brown particles in nests were considered positive (Fig. 1).

### Follow-up and statistical analysis

Information was collected on the 80 patients with LSCC by letter, telephone and outpatient follow-up visits. Follow-up continued to February 2012; the follow-up period was defined as from the date of the definitive diagnosis to the final visit or time of death. All data were analyzed using SPSS 17.0 software (SPSS Inc., Chicago, IL, USA). Correlation analyses used the Chi-square test. Kaplan–Meier survival curves were generated and log-rank tests were used to evaluate the differences between overall survival (OS) rates. A Cox regression model was applied for the multivariate analysis. The boundary for statistical significance was *P* < 0.05.

## Results

### Clinical features

The clinical data of 80 patients are summarized in Table 1. Overall, 75 males and five females were included in this study. Their ages ranged from 36 to 80 years, with a median of 60 years. The most commonly affected site was the glottic portion (*n* = 64; 80.0 %), followed by the supraglottic portion (*n* = 16; 20.0 %). All of the 80 patients underwent surgical resection without chemotherapy, and 46 cases received postoperative radiotherapy.

The patients had a survival time that ranged from 4 to 207 months (median, 73 months). Death occurred from the tumor in 47 cases, and the other 33 cases are still alive. Two patients developed distant metastases, one to the lung and the other to the brain.

### Therapeutic regimen

The treatment regimens were as follows: cordectomy (*n* = 23), cordectomy combined with radiotherapy (*n* = 16), partial laryngectomy (*n* = 6), partial laryngectomy combined with radiotherapy (*n* = 8), partial laryngectomy and neck dissection (*n* = 4), total laryngectomy (*n* = 13), total laryngectomy combined with radiotherapy (*n* = 1), total laryngectomy and neck dissection combined with radiotherapy (*n* = 7), primary radiotherapy and chemotherapy combined with neck dissection (*n* = 2).

The dose of radiotherapy was 40–68 Gy, which was delivered to the primary site in 27 cases, the neck in 2 cases and both the primary and neck regions in 5 cases. The operation margins were positive in eight cases and negative in 72 cases. Eight patients with positive margins were given postoperative radiotherapy, while 26 cases received radiotherapy because the lesions were extensive, although the margins were negative.

### Prognosis and follow-up

**Fig. 2:**
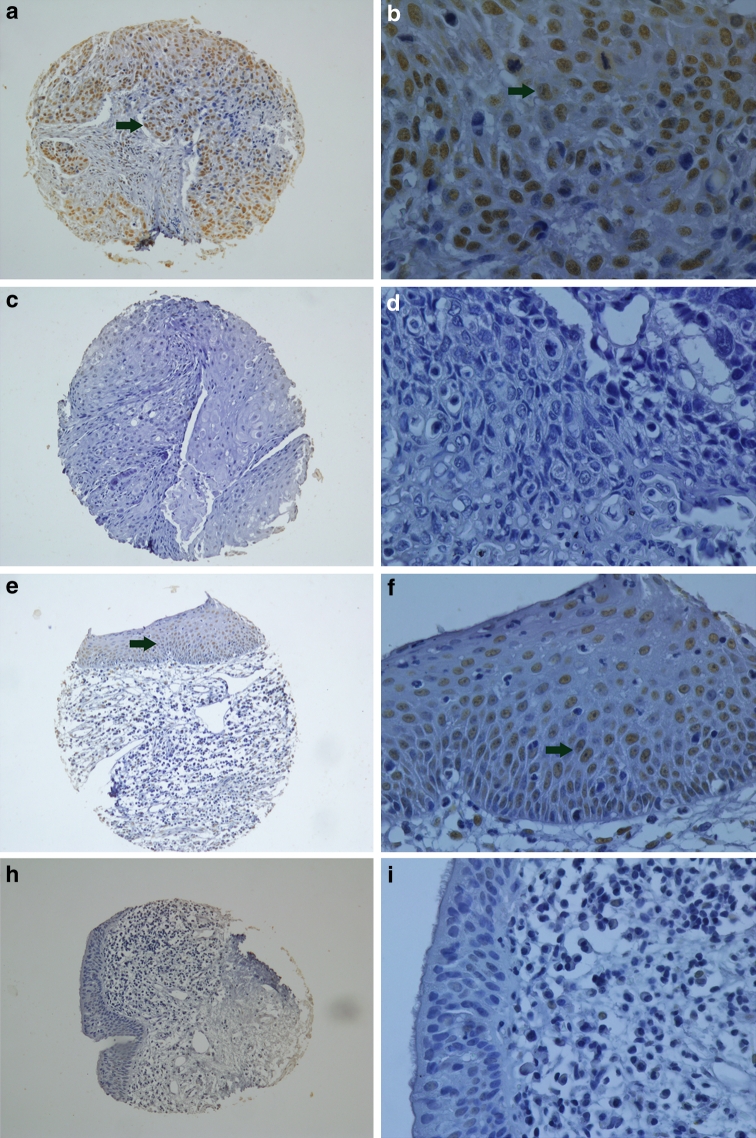
P300 expression according to immunohistochemical staining of LSCC samples. **a**, **b** A laryngeal squamous cell carcinoma (LSCC) case demonstrating a high expression level of P300 (*arrow*) detected in the nuclei of carcinoma cells (magnification: **a** ×100; **b** ×400); **c**, **d** Negative expression level of P300 detected in LSCC (magnification: **c** ×100; **d** ×400); **e**, **f** Positive expression level of P300 (*arrow*) detected in adjacent normal tissue of LSCC (magnification: **e** ×100; **f** ×400); **g**, **h** Negative expression level of P300 detected in adjacent normal tissue of LSCC (magnification: **g** ×100; **h** ×400)

**Fig. 3:**
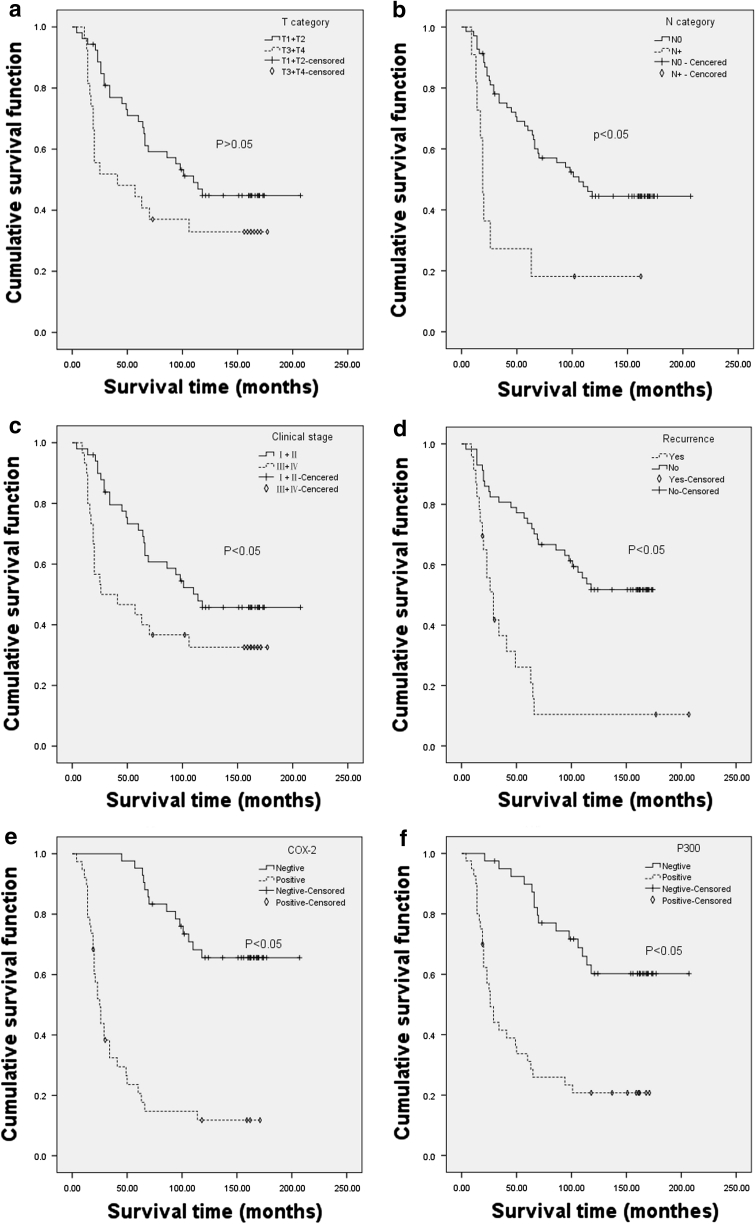
Kaplan–Meier survival analysis of clinicopathological factors associated with COX-2 and P300 expression in patients with LSCC (log-rank test). **a** Overall survival of the patients with different T categories; **b** overall survival of the patients with different N categories; **c** overall survival of the patients with different clinical staging; **d** overall survival of the patients with or without recurrence; **e** overall survival of the patients with or without P300 expression; **f** overall survival of the patients with or without COX-2 expression

The percentage of COX-2 and P300 expression in LSCC tissue were 47.5 and 50.0 %, respectively. Their expression in the adjacent normal tissues were 0.0 and 15.0 % for both (Figs. 1, 2). The expression levels of COX-2 and P300 were correlated with T stage, N stage, clinical stage, histological grade and recurrence (*P* < 0.05). COX-2 expression was correlated with P300 expression (*P* < 0.05) (Table 1). Univariate survival analysis showed that COX-2 and P300 expression levels, N category, clinical staging and recurrence factors were closely correlated with unfavorable survival (*P* < 0.05) (Table 2) (Fig. 3). Multivariate analysis showed that COX-2 expression, histological grade and recurrence were independent prognostic factors for LSCC (*P* < 0.05) (Table 3).

**Table 2:** Univariate survival analysis of 80 cases of LSCC

Factor	*N*	χ^2^	*P*
Gender			
Male	75	0.218	0.640
Female	5		
Age			
<60 years	42	1.048	0.306
≥60 years	38		
Medical history			
<12 months	61	-0.160	0.155
≥12 months	19		
Histology grade			
Well differentiated	39	3.308	0.191
Moderately differentiated	27		
Poorly differentiated	14		
T category			
T1 + T2	53	3.517	0.061
T3 + T4	27		
N category			
N0	69	10.919	**0.001**
N+	11		
Clinical staging			
I + II	50	4.526	**0.033**
III + IV	30		
Anatomic subsite of tumor			
S	16	1.294	0.255
G	64		
Recurrence			
Yes	23	22.833	**0.000**
No	57		
Resection margin			
Positive	8	3.105	0.078
Negative	72		
COX-2			
Positive	38	45.788	**0.000**
Negative	42		
P300			
Positive	40	23.777	**0.000**
Negative	40		

Bold values indicate statistical significance (*P* < 0.05)

*S* superglottic, *G* glottic

**Table 3:** Results of the Cox regression model for the analysis of laryngeal squamous cell carcinoma prognosis versus clinicopathological factors

Factor	*B*	SE	Wald	*df*	Sig.	Exp(*B*)	95.0 % CI Exp(*B*)	
							Low	High
Gender	−0.774	0.784	0.976	1	0.323	0.461	0.099	2.143
Age	0.034	0.018	3.514	1	0.061	1.035	0.998	1.073
Medical history	−0.041	0.025	2.692	1	0.101	0.960	0.914	1.008
Histology grade	−0.582	0.288	4.099	1	**0.043**	0.559	0.318	0.982
T category	0.204	0.726	0.079	1	0.779	1.226	0.295	5.090
N category	0.075	0.651	0.013	1	0.908	1.078	0.301	3.865
Clinical staging	0.052	0.378	0.019	1	0.890	1.054	0.502	2.212
Anatomic subsite of tumor	0.767	0.531	2.084	1	0.149	2.153	0.760	6.096
Recurrence	−1.118	0.386	8.375	1	**0.004**	0.327	0.153	0.697
Resection margin	1.480	0.779	3.610	1	0.057	4.393	0.954	20.223
Treatment modality	0.308	0.303	1.032	1	0.310	1.361	0.751	2.465
COX-2	2.413	0.449	28.835	1	**0.000**	11.164	4.628	26.931

Bold values indicate statistical significance (*P* < 0.05)

## Discussion

Laryngeal squamous cell carcinoma is one of the most common head and neck malignant tumors. We have observed that some patients that have the same histological grade and clinical stage have obviously different prognoses. Therefore, the search for biological indicators that are closely related to clinicopathological factors is very important.

### COX-2/P300 expression levels correlate with invasion and lymph node metastasis in LSCC

Recently, many studies have confirmed that COX-2 plays a role in many molecular tumorigenic mechanisms. For example, COX-2 can rapidly induce a response to tumor-promoting cytokines and growth factors through pathological pathways that affect mitosis, cell adhesion and immune monitoring. COX-2 has been therefore implicated in promoting tumorigenesis and cancer progression [3, 13].

Morita et al. [3] indicated that COX-2 promotes tumor lymphangiogenesis and lymph node metastasis in oral squamous cell carcinoma. Takatori et al. [4] investigated 228 cases of esophageal carcinoma patients and found that cases with COX-2 overexpression had deeper tumor invasion, more advanced clinical staging, poorer histological differentiation and poorer prognoses than those with low or no expression of COX-2. Sayar et al. [14] reported that the overexpression of COX-2 was a potentially important factor in the evolution of carcinogenesis in precancerous lesions and might be an indicator of carcinoma development. Sun et al. [15] found that the patients with pathological grade III and IV disease showed a higher level of COX-2 expression than those with grade I and II disease. The rate of COX-2 overexpression was higher in the cervical lymph node metastasis group than in the non-metastasis group. The expression of COX-2 has been closely associated with microvessel density (MVD) in LSCC.

P300, which is also known as KAT3B/EP300, is a transcriptional coactivator of various transcription factors. It is involved in a wide array of cellular activities, such as DNA repair, cell growth, differentiation, apoptosis and migration [16–19]. Liao et al. [20] confirmed that T staging and N staging were positively correlated with P300 expression. Li et al. [21] investigated the clinicopathological data of 240 patients with resectable esophageal carcinoma and confirmed that P300 overexpression was closely associated with poorly differentiated tumors and a higher TNM stage.

Our study showed that high levels of COX-2 and P300 were present in the patients with lymph node metastasis, advanced stage and poorly differentiated tumors than in those with no lymph node metastases, early staging and well or moderately differentiated tumors (*P* < 0.05). We hypothesize that COX-2 and P300 overexpression are correlated with invasion and metastasis in LSCC. However, we did not confirm that COX-2 and P300 were correlated with the anatomic subsite of the tumor (*P* > 0.05); COX-2/P300 overexpression rates were higher in the superglottic site group than in the glottic site group (75.0/62.5 vs. 56.3/46.8 %). Further research need to be carried out to confirm this finding.

### The relationship between COX-2/P300 expression and LSCC recurrence

Recurrence is an important factor that influences the prognosis of LSCC [22]. Grudzinski et al. [5] reported that the cases of breast cancer with COX-2-positive expression had shorter recurrence intervals of primary tumor than those with negative COX-2 expression, and COX-2 overexpression was an independent prognosis factor for primary recurrence. Meyer et al. [23] reported that COX-2 expression predicted a high risk of disease recurrence in patients with localized primary stage p T1-2 malignant melanoma. Isharwal et al. [10] detected that P300 was the highest risk marker for recurrent prostate carcinoma.

Our study showed that the recurrence rate of the group was 28.8 %, and COX-2 and P300 expressions were closely correlated with recurrence (*P* < 0.05). The COX-2/P300 expression rates were higher in the recurrence group than in the no recurrence group (82.6/78.3 vs. 33.3/38.6 %). From one aspect, this indicated that the COX-2 and P300 positive expression group was more susceptible to recurrence than the negative expression group. Therefore, we proposed that we should perform more close follow-up and adjuvant radiotherapy in cases with positive COX-2 and P300 expression. However, this hypothesis requires further animal experiments and clinical trials to prove the assumption. Even though three cases (3/20) of adjacent normal tissue were positive P300 expression, the case was not enough and the real clinical significance need to be further investigated.

### The relationship between COX-2/P300 expression and prognosis in LSCC

Most studies have reported that P300 and COX-2 overexpression are poor prognostic factors [4, 21, 24]. Chang et al. [24] investigated 82 cases of oropharyngeal squamous cell carcinoma and detected that cases with COX-2 expression had a worse prognosis than those with negative COX-2 expression. Li et al. [21] reported that esophageal carcinoma cases with P300 overexpression had a worse survival rate than those with lower expression (mean survival times were 56.9 and 80 months, respectively). However, different opinions exist regarding COX-2. Park et al. [25] showed that COX-2 expression was positive in 493/861 (57.3 %) of invasive tumors, but this marker alone was not related to survival outcome. However, [COX-2(+)/Ki-67(+)] tumors were significantly associated with unfavorable factors and worst survival outcomes, but the [COX-2(+)/Ki-67(−)] tumors showed significantly favorable parameters and better outcomes. COX-2(−)/Ki-67(any) showed an intermediate prognosis. Atula et al. [26] reported that COX-2 expression does not have any prognostic significance in advanced oral and pharyngeal squamous cell carcinoma, and therefore further studies should be carried out to verify the role of COX-2 in cancer invasion and metastasis.

Some scholars have proposed that the inhibition of P300 can prevent tumorigenesis [27, 28]. Xiao et al. [12] found that quercetin suppresses COX-2 expression mainly by inhibiting the P300-mediated acetylation of transactivators in human breast cancer cells. Takeuchi et al. [29] found that P300 expression reduced doxorubicin resistance in bladder cancer. These results indicate that P300 may be a promising molecular therapeutic target.

As P300 expression was positively related to COX-2 expression (*P* < 0.05), P300 factor was not introduced into the Cox regression model. Our study showed that COX-2 overexpression was an independent high-risk factor for prognosis, and the average survival time in COX-2 or P300 positive group was lower than that in COX-2 or P300 negative group (46/60 vs. 164/155 months). Our results were similar to other reports. According to our present data, we propose that we should pay more attention to the patients with COX-2 or P300 expression, with more radical resections and closer follow-up.

Our team has investigated the influence of COX-2 on a human tongue carcinoma cell line and found that siRNA-COX-2 significantly inhibited its proliferation and invasion [30]. However, the molecular interactions between P300 and COX-2 still require further study.

## Conclusion

The expression levels of COX-2 and P300 were increased in LSCC tissues. High expression levels of COX-2 and P300 indicated poor survival outcomes for patients with LSCC. The interactions between COX-2 and P300 in LSCC require further investigation, but COX-2 and P300 may become biological targets in the treatment of LSCC.

## Abbreviations

LSCC: Laryngeal squamous cell carcinoma
S: Superglottic
G: Glottic
